# Hydroxyethyl starch for perioperative goal-directed fluid therapy in 2020: a narrative review

**DOI:** 10.1186/s12871-020-01128-1

**Published:** 2020-08-20

**Authors:** Alexandre Joosten, Sean Coeckelenbergh, Brenton Alexander, Amélie Delaporte, Maxime Cannesson, Jacques Duranteau, Bernd Saugel, Jean-Louis Vincent, Philippe Van der Linden

**Affiliations:** 1 Department of Anesthesiology, Erasme Hospital, Université Libre de Bruxelles, Brussels, Belgium; 2 Department of Anesthesiology and Intensive Care, Hôpitaux Universitaires Paris-Sud, Université Paris-Sud, Université Paris-Saclay, Hôpital De Bicêtre, Assistance Publique Hôpitaux de Paris (AP-HP), Le Kremlin-Bicêtre, France; 3 Department of Anesthesiology & Perioperative Medicine, Bicêtre Hospital, 78, Rue du Général Leclerc, 94270 Le Kremlin-Bicêtre, France; 4 Department of Anesthesiology & Perioperative Care, University of California San Diego, San Diego, USA; 5 Department of Anesthesiology & Intensive Care, Marie Lannelongue Hospital, Paris, France; 6 Department of Anesthesiology & Perioperative Medicine, University of California Los Angeles, Los Angeles, USA; 7 Department of Anesthesiology, Center of Anesthesiology and Intensive Care Medicine, University Medical Center Hamburg-Eppendorf, Hamburg, Germany; 8 Outcomes Research Consortium, Cleveland, OH USA; 9 Department of Intensive Care, Erasme Hospital, Université Libre de Bruxelles, Brussels, Belgium; 10 Department of Anesthesiology, Brugmann Hospital, Université Libre de Bruxelles, Brussels, Belgium

**Keywords:** Colloid, Balanced crystalloids, Fluid responsiveness, Hemodynamic monitoring, Acute renal failure, Outcome

## Abstract

**Background:**

Perioperative fluid management – including the type, dose, and timing of administration –directly affects patient outcome after major surgery. The objective of fluid administration is to optimize intravascular fluid status to maintain adequate tissue perfusion. There is continuing controversy around the perioperative use of crystalloid versus colloid fluids. Unfortunately, the importance of fluid volume, which significantly influences the benefit-to-risk ratio of each chosen solution, has often been overlooked in this debate.

**Main text:**

The volume of fluid administered during the perioperative period can influence the incidence and severity of postoperative complications. Regrettably, there is still huge variability in fluid administration practices, both intra-and inter-individual, among clinicians. Goal-directed fluid therapy (GDFT), aimed at optimizing flow-related variables, has been demonstrated to have some clinical benefit and has been recommended by multiple professional societies. However, this approach has failed to achieve widespread adoption. A closed-loop fluid administration system designed to assist anesthesia providers in consistently applying GDFT strategies has recently been developed and tested. Such an approach may change the crystalloid versus colloid debate. Because colloid solutions have a more profound effect on intravascular volume and longer plasma persistence, their use in this more “controlled” context could be associated with a lower fluid balance, and potentially improved patient outcome. Additionally, most studies that have assessed the impact of a GDFT strategy on the outcome of high-risk surgical patients have used hydroxyethyl starch (HES) solutions in their protocols. Some of these studies have demonstrated beneficial effects, while none of them has reported severe complications.

**Conclusions:**

The type and volume of fluid used for perioperative management need to be individualized according to the patient’s hemodynamic status and clinical condition. The amount of fluid given should be guided by well-defined physiologic targets. Compliance with a predefined hemodynamic protocol may be optimized by using a computerized system. The type of fluid should also be individualized, as should any drug therapy, with careful consideration of timing and dose. It is our perspective that HES solutions remain a valid option for fluid therapy in the perioperative context because of their effects on blood volume and their reasonable benefit/risk profile.

## Background

Perioperative fluid therapy is a routine aspect of daily clinical practice for most anesthesiologists but remains a therapeutic challenge. One of the most complex aspects of perioperative fluid therapy is determining how much fluid to give each patient. Numerous observational studies have reported a strong association between both excessive and insufficient perioperative fluid administration and an increased risk of postoperative complications [1–6]. As it is difficult to predict or anticipate the volume of fluids a patient will need during surgery, several national and international societies recommend using goal-directed fluid therapy (GDFT) based on advanced hemodynamic monitoring in patients undergoing high-risk surgery [7–10]. GDFT has been promoted as helping to standardize fluid administration using recommended and validated protocols, thereby improving patient outcomes and decreasing costs [11–13]. Despite an abundance of literature on this topic, the volume of fluid that should be administered to achieve and maintain normovolemia is still the subject intense controversy.

The choice of intravenous fluid type has also been a subject of passionate debate, recently refueled by the publication of several large, prospective, randomized studies in different patient populations. These studies have demonstrated the impact of intravenous fluid solutions on patient outcomes, particularly in critically ill patients in the intensive care setting [14]. Although their results have created intense controversy, these studies have clearly demonstrated that the need for intravenous infusions may vary considerably in a given patient during the course of his/her clinical course [15]. They have also indicated that results observed in critically ill patients cannot be extrapolated to surgical patients. The R.O.S.E. conceptual model (Resuscitation, Optimization, Stabilization, Evacuation) condenses precisely a dynamic approach of fluid therapy allowing to maximize its benefits while reducing its harms [16]. Importantly, surgical patients receiving i.v fluids in the perioperative setting are typically in the “Optimization phase” and this specific category of patients will be discussed in the present review. Therefore, the goal of this narrative review article is to discuss the fundamentals of perioperative fluid therapy through four frequently asked questions regarding perioperative fluid therapy.

## Main text

### Question 1: should we administer fluids in a goal-directed fashion?

YES.

There is quite strong evidence to support the benefits of GDFT in high-risk patients undergoing major surgical procedures [9, 11]. Indeed, over the past 15 years, several meta-analyses of the impact of GDFT in patients undergoing moderate and high-risk surgeries have observed that GDFT improves outcome compared with routine care [8, 11, 17–20]. However, the studies included in these meta-analyses are highly heterogeneous, with different protocols, different physiologic endpoints, and different technologies to measure stroke volume and cardiac output. Interestingly, these studies demonstrate that patients in the GDFT groups also received highly variable volumes of fluids. While clinical pathways and protocols are not designed to eliminate all forms of variability, differences in individual care should be patient driven and not practitioner dependent. Adequate GDFT for patients undergoing major surgery requires that stroke volume or cardiac output are monitored to assess fluid responsiveness and that an algorithm is designed that will be applied by all members of the anesthesia team. Admittedly, some studies have not demonstrated a beneficial effect of GDFT on patient outcome [21–25]. However, these studies were mainly underpowered and/or conducted in relatively healthy patients with minimal fluid shift and blood loss [23, 24, 26–28]. Additionally, compliance to the study protocols must be examined closely when evaluating the results [29]. Pearse et al. nicely demonstrated that when a GDFT protocol is consistently applied, the treatment effect is strengthened [25]. Indeed, according to the authors of this study, “In the prespecified adherence-adjusted analysis conducted using established methods, the observed treatment effect was strengthened when the 65 patients whose care was non adherent were assumed to experience the same outcome as if they had been allocated to the alternative group (RR, 0.80; 95% CI, 0.61–0.99; *P* = 0.04)”. Another point of interest is that use of a GDFT protocol has never been associated with a deleterious effect for the patient. It is therefore not surprising that GDFT has been included in the national expert recommendations of several countries, including the United Kingdom and France [7, 10]. Obviously, some questions related to GDFT remain unanswered. What is the ideal endpoint? What is the best monitor to use when applying GDFT? What should be the ideal maintenance crystalloid infusion rate? What is the ideal “type” of fluid (crystalloid alone or a combination of crystalloid and colloid)? What is the ideal target patient population? Should GDFT protocols include inotropic support?

Most institutions and anesthesiology departments have written protocols and standardized pathways for management of severe perioperative bleeding, although “level 1A” evidence is mostly absent [30, 31]. Application of these protocols has demonstrated reduced variability in treatment and improved quality of care [32]. In our opinion, the same approach should be applied to hemodynamic and fluid management. In accordance with international guidelines, institutions should be encouraged to establish a written GDFT protocol. These GDFT strategies can rely on pulse pressure variation alone, enabling use in the absence of a cardiac output monitor and in clinical settings where more advanced monitoring is not available. As a result, implementing GDFT should not be associated with a large increase in costs.

### Question 2: is it possible to improve current GDFT?

YES.

Despite the development of minimally invasive monitoring devices and the simplification of GDFT protocols over the last decade [33, 34], clinicians’ compliance with the application of these protocols remains poor, ranging between 62 and 87%, even in ideal study conditions [9, 35]. Adherence of less than 50% to protocols is reported in daily practice across different medical specialties, but at least 80% adherence is required to observe improved clinical outcomes [36–38]. Effective management and application of GDFT algorithms often requires careful monitoring with frequent and repeated interventions by the clinician [34]. This can be particularly difficult for anesthesiologists who work in a stressful environment and are subject to numerous stimuli and distractions, all of which can decrease their attention and concentration.

**Fig. 1:**
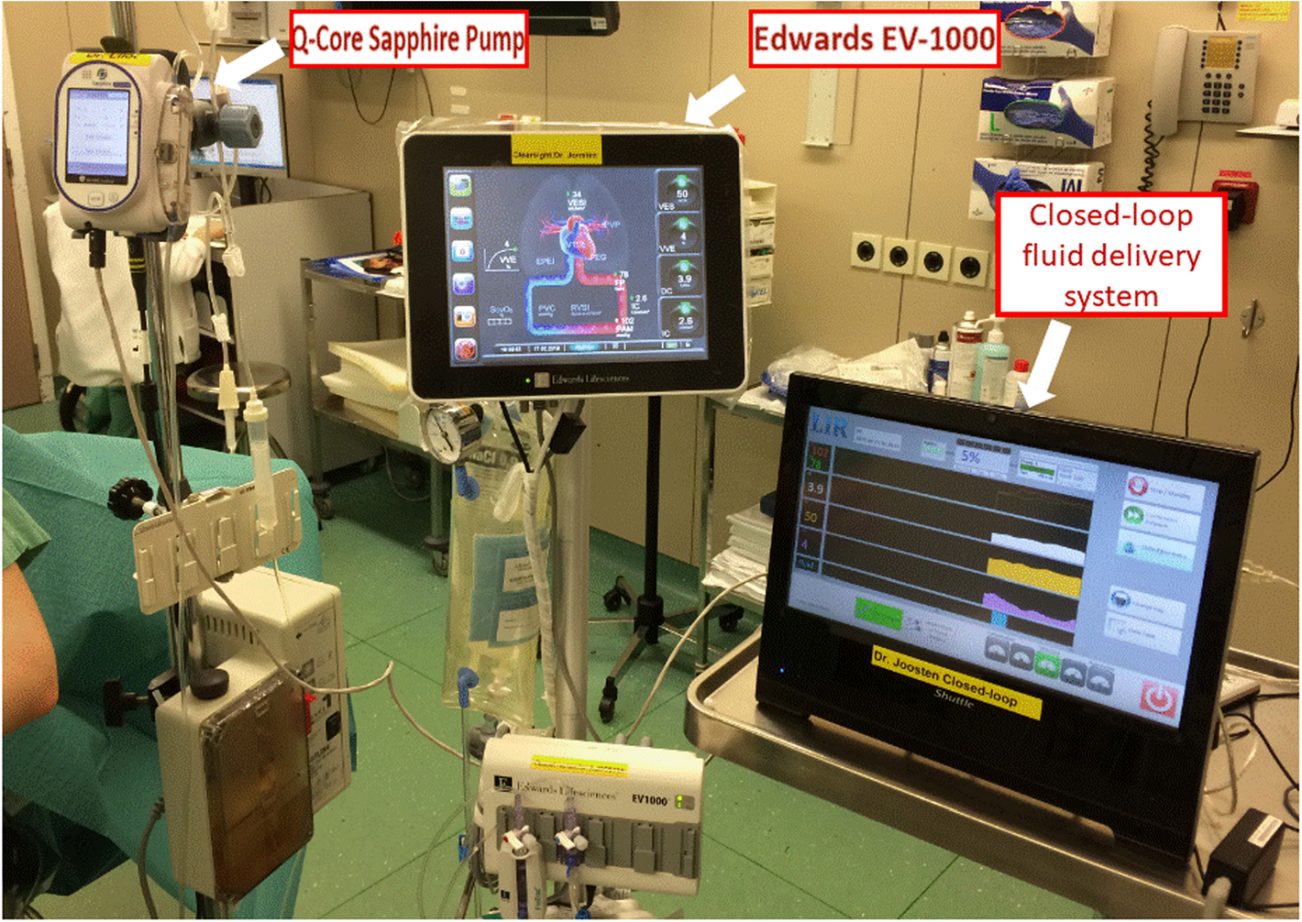
Closed-loop fluid management set-up. The closed-loop was connected with the EV1000 monitoring device with an analog-to-digital adapter connected to the EV1000 analog output device. The closed-loop software was run on a Shuttle X50 touchscreen PC. A Q-core Sapphire Multi-Therapy Infusion Pump (Q-Core, Netanya, Israel) was used to deliver mini fluid challenges of 100 ml and was linked to the closed-loop through a serial connection

In a recent prospective feasibility study, Menger et al. paired a second anesthesiologist, who was specifically dedicated to applying the written GDFT protocol, with the primary anesthesiologist in charge of the patient. With this “active clinical decision support system”, the authors observed a protocol compliance of about 85% [39]. Unfortunately, such an approach is very costly in terms of human resources, which limits its implementation [40]. Over the past decade, members of our team have developed a closed-loop administration system based on the simultaneous analysis of multiple advanced hemodynamic indices provided by a minimally-invasive hemodynamic monitoring device and controlled by a computer [27, 41–43] (Fig. 1). With this system, the administration of multiple fluid boluses is completely automated, requiring only minimal human intervention. We have demonstrated that implementation of this “computer system” resulted in less intraoperative time spent in a preload-dependent state (stroke volume variation > 13%) compared to a manually applied protocol, using both minimally invasive and noninvasive technologies [43, 44]. Implementation of these systems at the patient’s bedside has also been associated with better patient outcomes compared to routine care [45]. The software for this technology has now been implemented into the EV1000 monitoring device as a real-time clinical decision support system and is widely available for clinical use [46]. Of note, after the initial development of our closed-loop system for GDFT administration, our attention has now shifted to automated system allowing tight vasopressor infusion in order to be able to design a fully automated system for perioperative goal directed hemodynamic therapy, allowing the co-titration of fluid and vasopressors [47–52].

### Question 3: is there a place for a colloid solution in GDFT protocols?

YES.

**Fig. 2:**
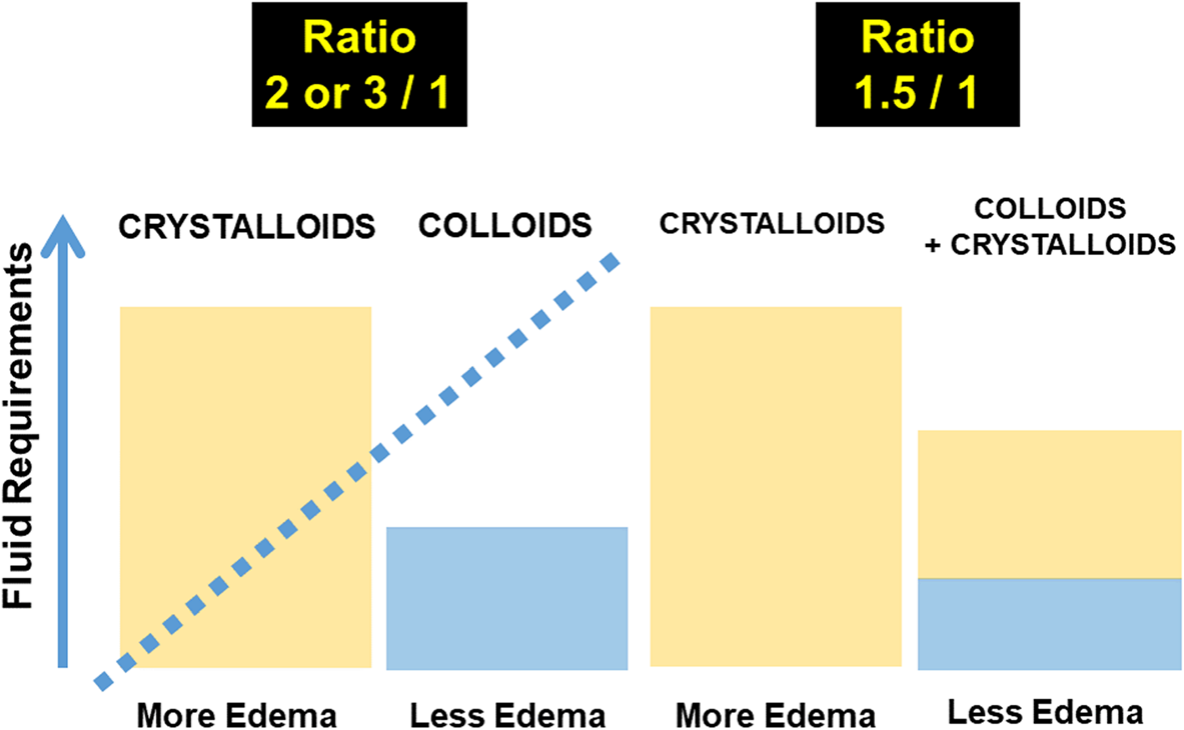
Comparison of the fluid requirements necessary for the optimization of the patient. The question is not to compare the administration of colloids versus crystalloids, but to compare crystalloids without or with a certain amount of colloids

From a physiological perspective, it seems obvious that colloid solutions have a role in GDFT protocols [53]. Colloid solutions remain longer intravascularly than do crystalloid solutions, continuing to create oncotic pressure, so theoretically they are associated with a decrease in the amount of fluid needed to achieve and maintain hemodynamic goals [54–57]. The exclusive use of crystalloid solutions, because of their lower volume effect and shorter intravascular persistence, is associated with greater volumes of fluid administration resulting in fluid overload and its potential complications in the perioperative period [57]. Indeed, compared to colloid solutions, greater crystalloid fluid volumes may be required to restore intravascular volume [58]. In an experimental study, Hiltebrand et al. showed that compared to a crystalloid solution (Ringer’s lactate), administration of HES boluses, guided by the measurement of venous oxygen saturation, reduced the total volume of fluid infused and, more importantly, improved microcirculatory blood flow and intestinal oxygen partial pressure after abdominal surgery [59]. Excessive fluid administration (mainly crystalloids) has been shown to increase the risk of intestinal tissue edema, leading not only to delayed resumption of intestinal activity and anastomotic leakage, but also to a risk of pulmonary edema and postoperative respiratory complications, all of which increase the hospital length of stay [6, 60]. The use of large volumes of isotonic saline (0.9% NaCl) also leads to an increased risk of hyperchloremic acidosis, which can lead to gastrointestinal and renal dysfunction, secondary to the vasoconstrictive properties of the chloride ion [61]. Importantly, volume effects of colloids have been demonstrated to be context sensitive [62]. Administration of iso-oncotic colloids (5% albumin or 6% hydroxyethyl starch) during acute bleeding when carefully maintaining intravascular normovolemia led to volume effects of more than 90% [63, 64]. In contrast, preoperative volume loading in a non-bleeding patient resulted in a volume effect of only 30–35%, two-third of the given bolus leaving the vascular space toward the interstitial compartment within minutes [65]. Colloids and crystalloids cannot be exchanged by simply adapting the amount [66]. Recently, Orbegozo Cortes et al. reviewed all studies comparing crystalloid and colloid solutions in all types of patients (medical, surgical, and trauma patients), many of which likely had altered vascular permeability [54]. They reported that greater volumes were required to achieve similar targets with crystalloid than with colloid solutions (estimated ratio: 1.50; 95% CI: 1.36–1.65). Figure 2 represents the comparison of the crystalloids vs colloids ratio extracted from the study from Orbegozo et al. in the context of an administration of crystalloids without or with a certain amount of colloids [54]. These results were confirmed by Annane et al. in the CRISTAL study [67]. Although this ratio would be expected to be greater than 1.5, it is important to note that none of these studies strictly compared a pure colloid with a pure crystalloid strategy as the colloid groups also always received some crystalloid infusion.

In the context of perioperative GDFT, there are very few clinical data evaluating the influence of the type of intravenous fluid used on outcome. Not surprisingly, there are currently no recommendations as to the type of fluid (crystalloids or colloids) that should be used to optimize a patient’s intravascular blood volume in the perioperative setting. It is interesting to note that 85% of the GDFT studies published in the literature used a colloid solution to optimize the patient’s stroke volume and cardiac output [53]. Most of these studies demonstrated a benefit in favor of the GDFT group versus the control group, which should encourage our academic community to continue examining the potential benefits of colloid use moving forward.

### Question 4: is there a place for hydroxyethyl starch solutions?

YES.

Albumin is the most frequently used colloid solution and is considered to be the colloid solution of choice. The main limitations to its use are cost and availability, which have led to the development of synthetic colloid solutions as alternatives. Among synthetic colloid, starches are by far the most studied solutions.

HES solutions are plant-based (corn or potato) colloid solutions derived from the enzymatic hydrolysis of starch. Their properties are defined by molecular weight, degree of substitution, C2/C6 ratio and concentration. They are available as either 0.9% saline or balanced crystalloid solutions. The most recent (third) generation of HES solutions has a lower molecular weight, which maintains oncotic effects while simultaneously decreasing adverse events (i.e., hemostatic alterations, renal failure, and pruritus). The maximum daily recommended dose is 30 ml/kg.

There is considerable controversy regarding the benefits and risks associated with the use of HES solutions. This controversy has stemmed from large multicenter studies in critically ill patients, and more specifically septic patients, which have reported adverse effects of HES solutions on mortality and/or renal function [68–70]. These studies have prompted the European Medicine Agency to restrict its use in the perioperative context. Several meta-analyses showed no association between HES administration and worse outcome in surgical patients [71, 72], so these concerns likely do not apply to short term intraoperative fluid expansion. Additionally, most studies that have demonstrated a benefit of GDFT over standard of care in high-risk surgical patients have used HES solutions to optimize stroke volume or cardiac output [53].

One small single-center, single-blinded randomized trial compared 5% albumin solution to 6% HES 130/0.4 solution used as part of a GDFT protocol in patients undergoing elective cystectomy [73]. There was no significant difference between the two groups with respect to the primary outcome, i.e., kidney function and kidney injury assessed up to postoperative day 90. Of note, the incidence of pruritus, evaluated by a questionnaire, was significantly higher in the albumin group.

Four double-blinded randomized studies have assessed the impact on patient outcome of HES 130/0.4 solution versus a crystalloid solution while standardizing the volume and timing of administration using a GDFT protocol [56, 57, 74, 75]. Yates et al. reported no benefit of HES solution over Ringer’s lactate (Hartmann) solution in terms of postoperative complications in 202 patients undergoing colorectal surgery [56]. However, in this monocenter study, 38% of patients in the crystalloid group received a rescue colloid solution (a gelatin) compared to 12% in the HES group. Interpretation of the results is thus challenging as this study actually compared two groups that received a combination of crystalloid and colloid solutions in different proportions. We observed that a HES-based GDFT was associated with a lower incidence of postoperative complications than a balanced crystalloid GDFT in 160 patients undergoing major abdominal surgery [57]. In a multicenter study (*N* = 1057), Kabon et al. reported that Doppler-guided intraoperative HES administration did not reduce a composite outcome of serious postoperative cardiac, pulmonary, infectious, gastrointestinal, renal and coagulation complications compared to lactated Ringer’s solution in patients undergoing moderate-to-high risk abdominal surgery [74]. In another multicenter study of 775 patients at increased risk of postoperative kidney injury after major abdominal surgery, Futier et al. reported that HES solution used according to a stroke volume-guided hemodynamic therapy algorithm did not reduce a composite outcome of death or major postoperative complications compared to isotonic saline [75]. However, more patients in the HES group developed mild acute kidney injury (*P* = .03) in the immediate postoperative period. In contrast, we did not observe any deleterious effect of HES solution on long-term (1 year) kidney function compared to a balanced crystalloid solution in our study population [76].

**Fig. 3:**
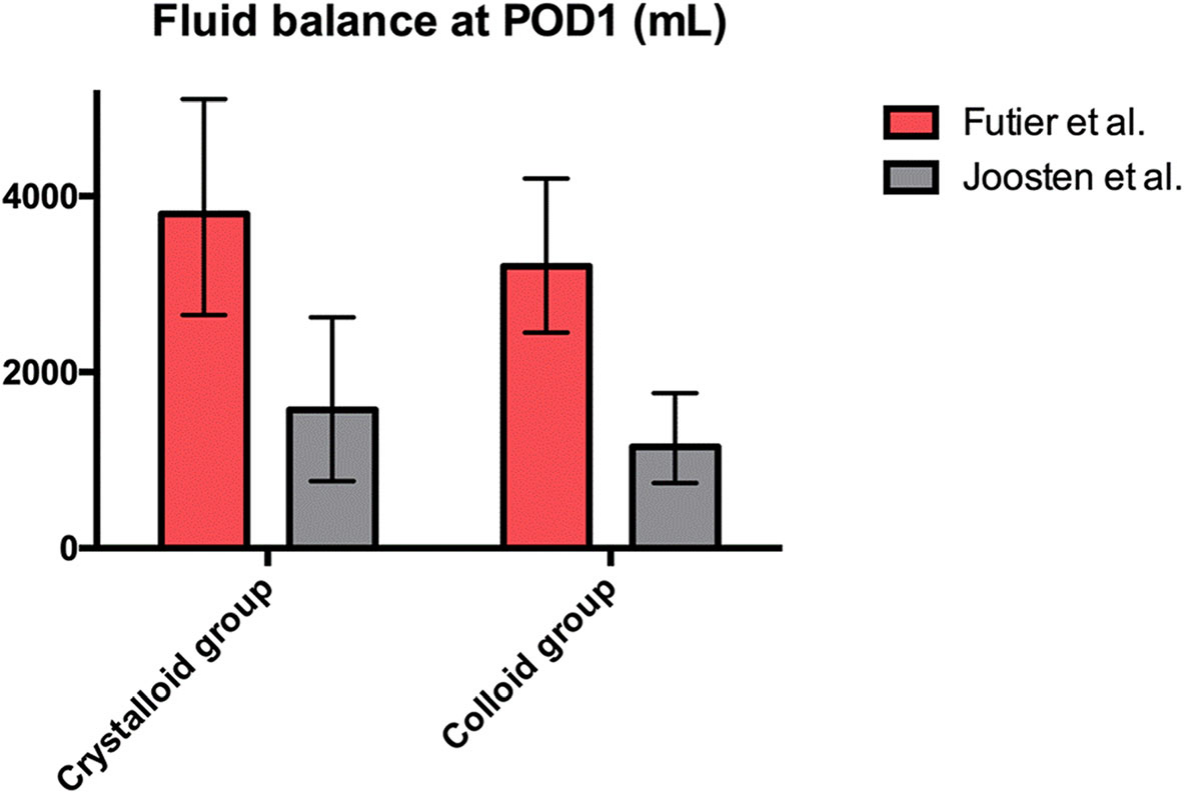
Comparison of fluid balance at postoperative day 1 (POD1) between the study of Futier et al vs Joosten et al

Not surprisingly, these four studies confirm what physiology tells us: to achieve a predefined hemodynamic target, a smaller volume of colloid solution, namely HES 130/0.4, is required, compared to crystalloid solution, resulting in a less positive intraoperative fluid balance. Of note, several studies have reported an association between positive perioperative fluid balance and worse outcome [77–80]. However, among the four studies cited above, our study reported a reduced incidence of postoperative complications with the use of HES solution, associated with a lower intraoperative fluid balance [57]. Interestingly, the major difference between our study and the three others is the protocol used to guide fluid administration. While the three other studies [68, 69, 71] used a pragmatic approach in which fluid boluses (250 ml fluid challenges) were administered manually by the clinician in charge of the patient in order to maximize stroke volume, we used a closed-loop delivery system in which small boluses (100 ml mini-fluid challenges) were administered automatically by a computer-controlled infusion pump with dedicated software that optimized stroke volume [70]. This system allowed strict standardization of fluid administration, increasing compliance with the protocol and improving the accuracy of implementation. The pragmatic approach used in the other studies, which reflects routine clinical practice, is inherently associated with lower protocol adherence and a high potential risk of protocol violations. When comparing our study to the study by Futier et al., the different strategies resulted in more “liberal” fluid administration in the Futier et al. study and a more “restrictive” approach in ours. However, the amount of HES solution infused in the colloid groups of the two studies was approximately the same (±1000 ml), whereas the total volume of crystalloid solution was much higher in the study by Futier et al. Consequently, cumulative net fluid balance on day one was also much more positive in this latter study (Fig. 3), which might explain the difference in the results reported in these two studies. In accordance with this hypothesis, fluid volume rather than fluid type may be responsible for the divergent results seen across the four studies. Importantly, the study of Joosten et al. [57] included the smaller number of patients compared to the three other ones [56, 74, 75]; their results need to be therefore confirmed. Two large randomized controlled trials (TETHYS trial [*N* = 350] in trauma patients and PHOENICS trial [*N* = 2280] in elective abdominal surgical patients) are on the way. They should clearly help to precise the benefit to risk ratio associated with the use of HES in the two specific contexts. In the final debate, the price should be taken into account as HES solutions are much more expensive than crystalloid solutions, but significantly cheaper than albumin solutions in most European countries.

## Conclusion

In the perioperative setting, not only the type of fluid but also the volume administered can impact a patient’s outcome following major surgery. Fluid volume should be guided by predefined physiologic targets, using an individualized hemodynamic algorithm. Clinician compliance with such protocols can be improved by using automated closed-loop systems that enable automation of some of the simple but restrictive therapeutic tasks. It is of prime importance that intravenous fluids be administered with the same care as any other drug, with strict indications and contraindications and precautions with regard to potential adverse effects. Finally, perioperative colloid solutions, and in particular HES solutions, may have a place in optimizing a patient’s hemodynamic status while limiting the total volume of fluid administered. Large multicenter studies that assess the impact of crystalloids vs colloids and that apply a strict approach that ensures high protocol compliance, for example with a closed-loop system, are needed.

## Data Availability

NA

## Abbreviations

GDFT: Goal directed fluid therapy
HES: Hydroxyethyl starch
